# Histology correlated adaptive optics polarisation sensitive optical coherence tomography

**DOI:** 10.1364/BOE.590238

**Published:** 2026-05-19

**Authors:** Thomas J. Smart, Bruno Charbit, Ziqi Zhou, Yuan Tian, Arman Athwal, Jun Song, Myeong Jin Ju, Colin J. Chu, Marinko V. Sarunic

**Affiliations:** 1 Institute of Ophthalmology, University College London, London EC1V 9EL, United Kingdom; 2 Department of Medical Physics and Biomedical Engineering, University College London, London WC1E 6BT, UK; 3 School of Biomedical Engineering, University of British Columbia, Vancouver, BC, Canada; 4 Department of Ophthalmology and Visual Sciences, University of British Columbia, Vancouver, BC, Canada; 5 Moorfields Eye Hospital, London, London EC1V 2PD, United Kingdom; 6 School of Engineering Science, Simon Fraser University, Burnaby BC V5A 1S6, Canada

## Abstract

Adaptive optics polarisation sensitive optical coherence tomography (AO-PS-OCT) provides high-resolution and tissue-specific contrast for the study of retinal pathology. We used our recently developed AO-PS-OCT system to acquire longitudinal *in-vivo* images of laser injury in the mouse retina over a ten-day period. Hyper-reflective foci (HRF) were observed in both reflectance and PS-OCT modalities. Spatial registration with immunohistochemistry showed co-localisation of HRF in our PS-OCT modality with IB4-positive myeloid cells and probable pigment in the confocal transmission channel, suggesting uptake of melanin-containing RPE debris as a source of PS contrast. These results demonstrate the potential of AO-PS-OCT for discrimination of HRF *in-vivo.*

## 1. Introduction

Optical coherence tomography (OCT) is a non-invasive volumetric imaging modality. Since its introduction some 35 years ago [1], OCT has become a widely employed tool in ophthalmology [2]. Hyper-reflective foci (HRF) in retinal OCT images, a term defined as hyperreflective dots or roundish lesions within the retinal layers [3], are a biomarker of progression, treatment response and prognosis in several retinal diseases, such as age-related macular degeneration (AMD) [4], diabetic retinopathy [5] and uveitis [6]. However, the molecular content of these features is not well understood. OCT cannot directly provide molecular information and the clinicopathological correlate of HRF remains uncertain, ranging from lipid material, migrating retinal pigment epithelium (RPE) cells, macrophages/microglia, and degenerated photoreceptor cells [3,7,8].

Image contrast in traditional OCT is based on the intensity of light backscattered from the sample. Additional mechanisms of contrast have been developed, including polarisation-sensitive OCT (PS-OCT) which takes advantage of changes in the polarisation state of light scattered by the sample to derive a tissue-specific contrast mechanism [9]. Most structures in the eye do not change the polarisation state of light upon interaction. However, certain ocular structures are birefringent, and others are depolarising. Birefringent materials cause a relative retardation of orthogonal polarisation states, whilst depolarising materials scramble the polarisation state of light. Depolarisation can be caused by multiple scattering or by scattering at non-spherical particles [10]. Broadly, it is fibrotic materials (e.g. the cornea, the retinal nerve fibre layer, the sclera) that cause birefringence [11] and pigmented materials (e.g. melanin in the retinal pigment epithelium) which cause depolarisation [12]. PS-OCT has the potential to provide novel information in the setting of retinal pathology. For example, the ability to detect depolarising structures may provide information about the molecular content of HRF that allows for *in-vivo* discrimination. Here, we demonstrate high-resolution PS-OCT imaging of HRF by combining PS-OCT with adaptive optics (AO).

AO detects and compensates for aberrations present in the eye of a subject that cause degradation of image quality. Aberrations are typically compensated by a deformable mirror placed in the optical path of the imaging system. AO has previously been combined with OCT to enhance image resolution [13] and several groups have investigated the combination of AO with PS-OCT. Cense *et al.* combined AO with PS-OCT for human imaging and found improved signal to noise, smaller speckle size and increased lateral resolution compared to a system without AO [14]. Recently, Kurokawa *et al.* described an AO-enabled polarisation-sensitive human imaging system designed to detect early signs of neurodegenerative diseases such as glaucoma [15]. Our groups have previously performed PS-OCT imaging in mice, with only low-order aberration correction [16,17].

Several groups have presented preclinical OCT studies that correlate HRF with cellular markers in rodent models. Fan *et. al.* monitored HRF in the mouse following optic nerve crush injury and verified HRF as inflammatory cells with immunohistochemistry (IHC) [18]. Nat Nor *et. al.* reported characteristics of HRF in a rat model of diabetic retinopathy and showed that HRF corresponded to clusters of activated microglia [19]. Miller *et. al.* used OCT and scanning laser ophthalmoscopy to quantify the distribution and dynamics of microglia following laser injury. Their results showed migration of microglia and Müller cells to the damage site, as well as increased scattering from photoreceptors following injury [20].

In this report, we demonstrate the integration of PS contrast with our multimodality AO small animal retinal imaging system [21]. We present *in-vivo* images of a laser injury lesion over a ten-day period with higher-order aberration correction using our recently developed AO-PS-OCT system. AO-PS-OCT enables high-resolution, tissue-specific visualisation of histologic features such as HRF. We correlate of our *in-vivo* images with *ex-vivo* IHC to elucidate the source of contrast in our polarisation-sensitive images.

## 2. Methods

### 2.1. Polarisation-sensitive optical coherence tomography

The instrument used here is based on a custom-built small animal retinal imaging system that has been described in depth elsewhere [21,22]. In brief, the system integrates image-guided sensor-less adaptive optics with a fibre-based near-infrared spectral-domain OCT scheme. The light source is a superluminescent diode (SLD) with a central wavelength of 840 nm and a full-width half-maximum of 90 nm. The sample arm contains a variable lens (VL) that allows the position of the beam waist to be scanned in depth across different layers of the retina. A 69-actuator deformable mirror (DM) is used for correction of ocular aberrations. A pair of galvanometer mirrors scan the beam laterally across the region of interest in the retina. The VL, DM and GS are optically conjugated by lens-based telescopes with the pupil plane. A-scans are acquired from the two spectrometers simultaneously at a rate of 100kHz, with 500 a-scans per B-scan, and 400 B-scans per volume. The field of view is approximately 500 µm x 500 µm. At each location of the scan, two B-scans are captured to permit OCTA calculation, allowing PS-OCT and OCTA to be acquired simultaneously over a 4 second period. Alignment of the two spectrometers was carried out numerically [23]. The sensor-less AO algorithm has been described elsewhere [21]. Here, we optimised 21 Zernike modes using an image metric calculated on an *en-face* maximum intensity projection of the deep capillary plexus.

**Fig. 1.**
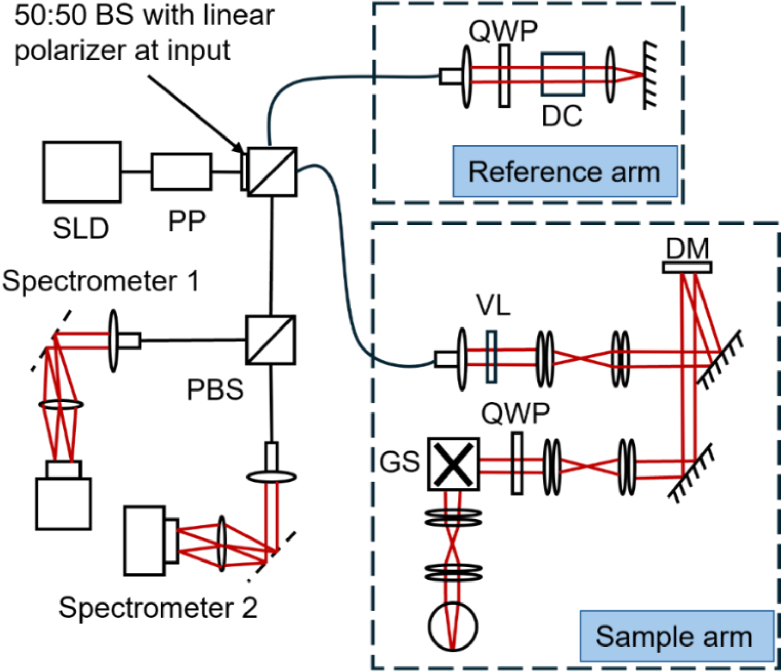
Schematic of optical system. Superluminescent diode, SLD; polarisation paddle, PP; beam splitter, BS; quarter wave plate, QWP; dispersion compensation block, DC; variable lens, VL; deformable mirror, DM; galvanometer scanners, GM; polarising beam splitter, PBS. Diagram not to scale.

Recently, we have modified our system to provide PS contrast (see Fig. 1). The PS-OCT interferometer is based on the system described in [24]. Light from the SLD is split into sample and reference arms by a non-polarising beam splitter. In the sample arm, a quarter wave plate (QWP) is oriented at 45^0^ to provide circularly polarised light at the cornea. In the reference arm, a QWP is oriented at 22.5^0^ to provide balanced power in the two spectrometers. Light from the two arms is recombined at the beam splitter and launched into a polarisation-maintaining fibre. The light is subsequently split into orthogonal polarisation states by a polarising beam splitter and analysed on two independent spectrometers.

### 2.2. OCT post-processing

Following performance of the usual OCT post-processing steps (DC-subtraction, re-mapping from λ to k space, dispersion compensation), OCT volumes were processed to provide reflectance and polarisation-sensitive contrast. The degree of polarisation uniformity (DOPU) [25] was calculated following the methods set out in [16] and [26]. Local spatial averaging was applied over a 3 × 4 pixels kernel (approximately 3 µm x 4 µm) in each B-scan. Low DOPU values imply the presence of depolarising material whereas high DOPU values imply a lack of depolarising material. OCT angiography (OCTA) was calculated on twice repeated B-scans using the speckle-variance method [27,28]. The time-course images shown below are the average of at least 30 volumes acquired at each time point. Volumes acquired at different time points were laterally registered based on the OCTA signal from the deep capillary plexus to ensure that the same lesion is compared at each time point. *En-face* OCT reflectance images were generated by taking a maximum intensity projection across several slices. DOPU *en-face* images were generated by taking minimum intensity projections.

### 2.3. Mouse husbandry and retinal photocoagulation model

Cx3cr1^GFP^Ccr2^RFP^ transgenic mice (Jackson Laboratory, strain no. 032127; RRID: IMSR_JAX:032127) were used to generate time-course images. The laser injuries in this study were performed on Cx3cr1^GFP^Ccr2^RFP^ transgenic mice, in which monocytes express red fluorescent protein and microglia express green fluorescent protein. Cx3cr1^GFP^Ccr2^RFP^ mice were chosen for our experiment as microglia are a likely component of HRF. Cx3cr1^GFP^Ccr2^RFP^ mice are derived from a C57BL/6 genetic background.C57BL/6J mice (Charles River, strain no. 027; RRID: IMSR_CRL:027) and albino (Charles River, strain number 022, Crl:CD1(ICR)) mice were used for validation. Mice were maintained in the Biological Research Unit at the Institute of Ophthalmology, University College London. All procedures were performed under a UK Home Office Project Licence (PP1506797), in accordance with the Animals (Scientific Procedures) Act 1986, and adhered to the ARVO Statement for the Use of Animals in Ophthalmic and Vision Research. Laser injury and time course imaging was performed on three mice. Two laser injuries were induced on each mouse retina using the Phoenix MICRON 5 *in-vivo* Image-Guided Laser system with a power of 200 mW and a duration of 0.07 s. Approximately 1-hour after *in-vivo* imaging on day 10, mice underwent cardiac perfusion [29] with 2% paraformaldehyde (PFA) before their eyes were removed and transferred in 4% PFA for 1-hour. Retinal tissues were then fixed overnight at room temperature (RT) in a 1:4 dilution of BD Cytofix (BD Biosciences).

### 2.4. Immunohistochemistry

Eyes were dissected to extract the retina, then blocked following standard IHC protocol. The samples were stained at room temperature for 72-hours under agitation, using the antibody mix of Hoechst 33342 (1:500; Cat. no. H3570, ThermoFisher), Collagen IV (1:100; Cat. no. ab34710, Abcam) and Isolectin B4 AF594 (1:200; Cat. no. I21413, ThermoFisher). Collagen IV was used as a marker for retinal vasculature. Isolectin B4 (IB4) stains endothelial cells, which line the interior of blood vessels. In inflammatory conditions, IB4 also stains activated microglia. Secondary antibody (1:500; Donkey anti-Rabbit IgG AF532, cat. no. A-11009, ThermoFisher) was added after a series of 3x 30 minutes washes with PBS and incubated for 48 hours at room temperature under agitation. Tissues were post-fixed with 1%-PFA for 15 minutes at RT then whole retinas were mounted on Corning frosted microscope slides (Scientific Laboratories Supplies) and cleared with Clearing-enhanced 3D (Ce3D, BioLegend) overnight at RT. Leica TCS SP8 HyVolution (Leica Microsystems) confocal microscope with 20x objective and 2X optical zoom was used to image the stained samples. AF488 channel was captured to visualize Cx3Cr1GFP-expressing cells and transmitted light imaging was used to identify pigmented structures.

### 2.5. Registration procedure

Correlation of fiducial markers from *in-vivo* OCT images and *ex-vivo* IHC images enables mapping of the cellular information provided by IHC onto the structural information provided by OCT. The retinal vasculature was used as a set of fiducial markers for correlation between modalities. Collagen IV-stained vasculature in the IHC images was registered to the OCTA signal from corresponding regions of the retina. Resident immune cells such as microglia that are visualised in IHC can then be indirectly registered with the OCT signal by applying the displacement field provided by registration of the vasculature, potentially leading to cellular phenotyping of OCT structures. Registration was performed in two steps: firstly, an affine transformation was applied to the collagen IV channel, calculated on the basis of cardinal points selected in both images. Secondly, a non-rigid deformation was automatically calculated and applied to the rigidly registered image. The deformation matrices calculated for the collagen confocal channel were subsequently applied to the other confocal channels.

## 3. Results

### 3.1. AO-PS-OCT system validation

**Fig. 2.**
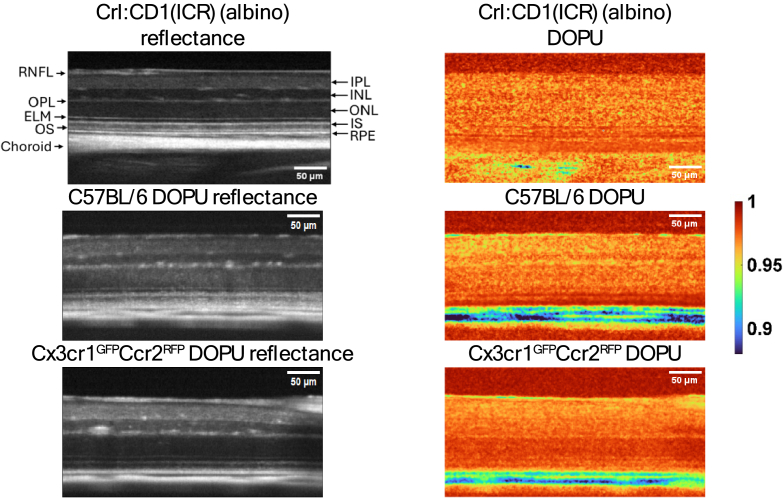
Top row: log-scale reflectance and DOPU of albino mouse retina. Second row: log-scale reflectance and DOPU of C57BL/6 mouse retina. Third row: log-scale reflectance and DOPU of Cx3cr1^GFP^Ccr2^RFP^ mouse retina. Retinal layers as indicated on reflectance image in top row: retinal nerve fibre layer (RNFL); inner plexiform layer (IPL); inner nuclear layer (INL); outer plexiform layer (OPL); outer nuclear layer (ONL); external limiting membrane (ELM); photoreceptor outer segments (OS); photoreceptor inner segments (IS); retinal pigment epithelium (RPE); choroid.

The efficacy of the polarisation-based contrast mechanism was demonstrated by comparing DOPU images of an albino mouse, which lacks melanin pigment, with pigmented mice (C57BL/6 and Cx3cr1^GFP^Ccr2^RFP^) - see Fig. 2. The DOPU-based contrast clearly highlights the RPE and choroid in the pigmented mice whereas these layers do not provide contrast in the albino mouse. The lack of pigment in the albino mouse allows for deeper penetration of light, revealing extra-orbital features in both the intensity and PS images, consistent with the findings of [30]. We compare PS-OCT images from a Cx3cr1^GFP^Ccr2^RFP^ mouse and C57BL/6 mouse in Fig. 2 and do not observe any marked differences in the DOPU-based contrast.

### 3.2. Time course imaging of laser injury in mouse retina

**Fig. 3.**
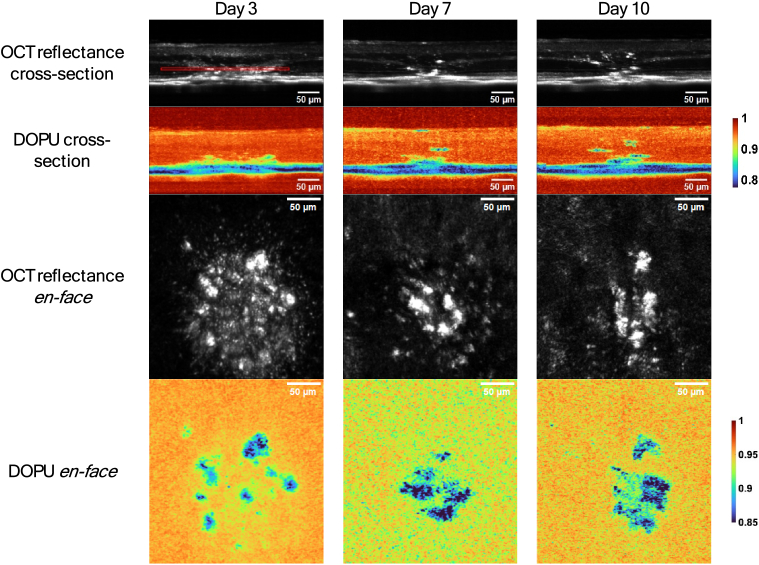
Cross-sectional and en-face time-course images of a representative laser lesion in the same mouse retina acquired 3 days, 7 days and 10 days after injury (mouse 1). Red box in cross-sectional image at day 3 represents the region over which projections were taken. Scale bar limits have been adjusted for en face DOPU images to highlight HRF.

Following validation, we used our AO-PS-OCT to study laser lesions in the mouse retina. Note that photocoagulation was performed only to form focal photoreceptor damage in the outer retina, rather than to induce choroidal neovascularisation. Laser injury was implemented on day 0 in three mice. *In-vivo* retinal imaging took place on days 3, 7 and 10. The captured OCT volumes were processed to provide reflectance and polarisation-sensitive contrast. Figure 3 shows cross-sectional and *en-face* images of a single lesion in a single mouse at each time point (we label this mouse ‘mouse 1’ in the following figures). *En-face* images are projections across several slices within the outer nuclear layer (ONL). The red box in the day 3 cross-sectional image indicates the slices over which the projections were taken, as well as the extent of the *x,y* plane. Many, but not all, of the hyperreflective features in the DOPU images can be matched to features in the OCT reflectance images.

Three days after injury, we observe HRF close to the RPE in the cross-sectional images in both contrast mechanisms. As time progresses, we observe a reduction in the number of HRF and a migration towards the inner retina. At day 3, we also observe dispersed hyperreflective signals between the outer plexiform layer and the photoreceptor layer in the OCT reflectance image. This feature contracts and becomes less reflective over time, which is consistent with the observations made in [31]. We also see increased thickness of the outer retina over time. This is particularly apparent in the DOPU images. The *en-face* images from the ONL likewise illustrate a reduction in the number of HRF over time as well. We also observe a coalescence of the HRF over time and an increase in size of individual HRF.

To better understand the origin of the DOPU contrast of HRF in these images, spatial correlation of *in vivo* and *ex vivo* imaging was performed and is described in the following section.

### 3.3. Correlation of in-vivo imaging with ex-vivo immunohistochemistry imaging

Following longitudinal *in-vivo* imaging, the mice were swiftly sacrificed and e*x-vivo* IHC imaging of the retinas was carried out as described in section 2.4. Collagen IV was used to label retinal vasculature basement membranes and isolectin B4 (IB4) was used to label endothelial cells and activated microglia.

**Fig. 4.**
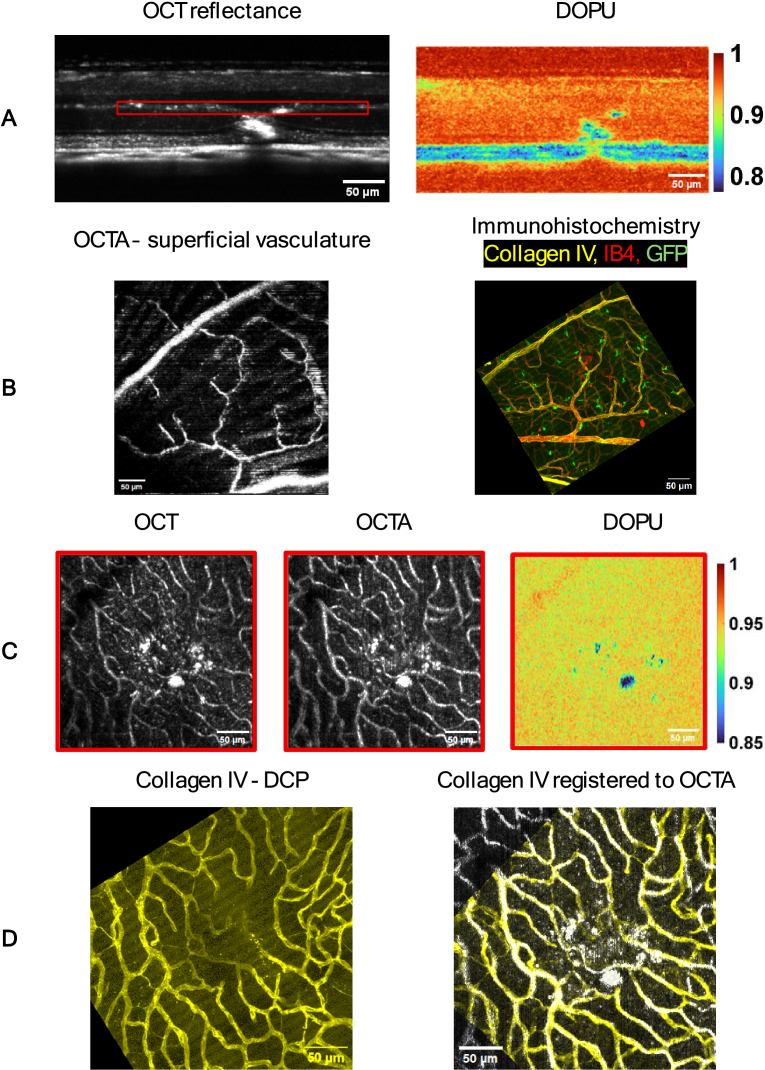
**A:** cross-sectional images of laser lesion 10 days after injury in OCT reflectance and DOPU modalities in mouse 2. **B:** OCTA projection of superficial vasculature, above site of lesion; IHC projection the same region. **C:** OCT reflectance, OCTA and DOPU projections at the deep capillary plexus (DCP); projections taken over region indicated in OCT reflectance image in panel A. **D:** collagen IV IHC projection of DCP; collagen IV registered to OCTA.

Figure 4 summarises the process of correlating of *in-vivo* and *ex-vivo* images. SubFig. 4(A) shows *in-vivo* cross-sectional images of the lesion at day 10. Note that the images shown in Fig. 4 are from a different mouse to those shown in Fig. 3 (we label this mouse ‘mouse 2’ in the following figures). 4B shows the OCTA signal from the superficial vasculature above the lesion and a maximum intensity projection of the *ex-vivo* retinal tissue at the same location from IHC imaging. The collagen IV channel is shown in yellow, IB4 in red, and GFP in green. The distinctive branching structure of the superficial vasculature is apparent in both the *in-vivo* OCTA and the *ex-vivo* collagen IV. 4C shows *en-face* projections of the deep capillary plexus (DCP) in OCT reflectance, OCTA and DOPU contrast. The three modalities all show a hyperreflective feature situated in the lower half of region of interest. This feature may also be observed in the cross-sectional images as a hyperreflective feature in the DCP. We believe the appearance of this feature in OCTA to be an artefact, rather than indicating flow [32]. 4D shows an *en-face* projection of the DCP in the collagen IV channel, as well as the result of registration of the collagen IV signal to OCTA signal.

**Fig. 5.**
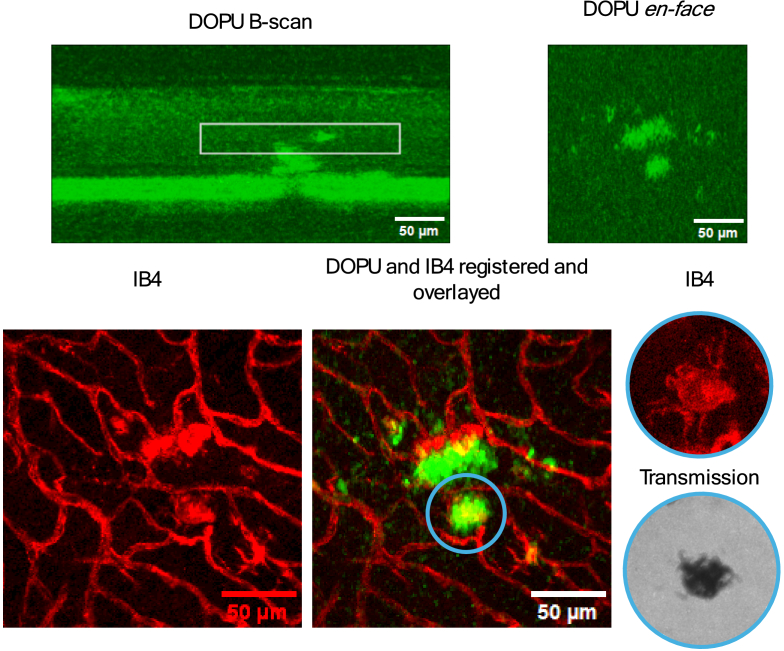
Correlation of imaging modalities in mouse 2. The top row shows DOPU B-scan and an en-face projection of the region outlined in the B-scan (using a green colour map, for purposes of overlay). The second row shows IB4 from deep capillary plexus at lesion site; overlay of IB4 on DOPU after registration; magnified images from IB4 and confocal transmission channels from within blue circle.

Figure 5 shows the results of correlating the IHC images with the DOPU signal. The IB4 channel is shown in red and the DOPU signal is now displayed with a green colour map to allow for visualisation of co-localisation. The images are from the same location as shown in Fig. 4. The registered IB4 channel is overlayed on the DOPU image. We see co-localisation of a hyperreflective DOPU feature with an IB4-labelled cell - circled in blue. We presume the IB4-labelled cell to be a myeloid cell. A magnified image of the presumptive myeloid cell is shown on the right-hand side. Also shown is the signal from the confocal transmission channel at the same location.

**Fig. 6.**
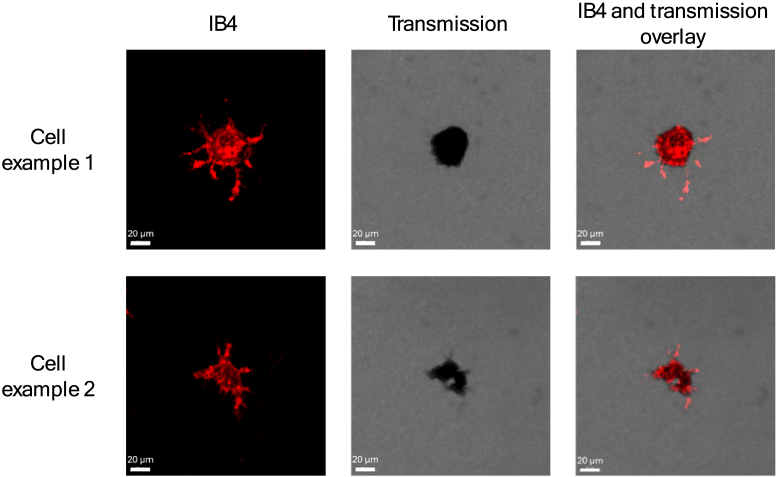
Examples of co-localisation of IB4-labelled cells and likely pigmented material in the confocal transmission channel

Dark regions in the transmission channel may correspond to pigmented material. This co-localisation of potential pigment and immune cells may suggest that pigment has been internalised by the cells. We see many other instances of co-localisation between IB4-labelled cells and pigmented features in the transmission channel, two examples of which are shown in Fig. 6. Given that the DOPU signal is also aligned with the cell, it is possible that internalised pigment is the source of DOPU contrast in this hyperreflective feature. The hyperreflective feature is also observed in the OCT reflectance image. Melanin is well-known to provide hyperreflectivity in OCT reflectance images [33]. Supplement 1 Figure S1 provides an example of a HRF in the OCT reflectance channel with a low contrast counterpart in the DOPU contrast. The HRF is co-localised with an IB4-labelled cell, but there is no accompanying dark feature in the transmission channel.

Other potential contributors to the DOPU signal include microglia, Müller cells, and damaged photoreceptor nuclei, all of which Miller *et. al.* showed to be present at the site of injury in period following injury [20], as well as collapsing material from the vasculature. The OCT reflectance image in Fig. 4(A) shows damage to the OPL, raising the question as to whether endothelial material from the vasculature could contribute to the IB4-labelled structures. Whilst it is possible for small amounts of ruptured endothelial antigen to be dispersed in the tissue, there would need to be extensive damage and yet Collagen IV shows deep plexus is largely intact. The morphology is also too focal and rounded for this to be the most likely observation, but we cannot dismiss this as a possibility.

## 4. Discussion and conclusion

We have developed an adaptive-optics-enabled polarisation-sensitive OCT system for small animal imaging. In this paper we have demonstrated the abilities of our AO-PS-OCT system by performing longitudinal imaging of laser-induced injuries in the mouse retina. We imaged three mice over a 10-day period following laser injury. Aberrations were corrected up to 21 Zernike modes. We were able to detect small highly reflective foci (HRF) in the inner retina both in traditional reflectance-based OCT contrast and polarisation-sensitive DOPU-based contrast.

We studied the dynamics of detected HRF over several days and observed a reduction in their number as well as migration towards the inner retina. Most but not all HRF visible in DOPU have counterparts in intensity-based OCT contrast. Detection of these features in the PS-OCT modality raised the question of what causes their polarisation-sensitive contrast. To shed light on this question, we performed *ex-vivo* IHC confocal imaging and spatially registered these images with our *in-vivo* images.

Following registration, we observed strong co-localisation of a HRF in the DOPU signal with an IB4-labelled myeloid cell and a pigmented structure detected in the confocal transmission. In the IHC imaging we found many instances of IB4-labelled cells co-localised with pigmented structures in the confocal transmission channel. Altogether, this may suggest that myeloid cells internalise pigmented RPE debris following laser injury to the outer retina, and that these pigment-containing myeloid cells provide a source of DOPU contrast for the observed HRF. As noted, other sources of DOPU contrast may include microglia, Müller cells, damaged photoreceptors and collapsing material from the vasculature. HRF in human OCT imaging are seen in a number of retinal diseases, but their cause is not well understood. In some cases, clinicopathology attributes HRF to migratory RPE cells. The ability of AO-PS-OCT to detect depolarising material may allow HRF to be discriminated in-vivo.

The primary limitation of this work is the small numbers of mice and HRF imaged. The time taken between *in-vivo* imaging and tissue fixation (on the order of one hour) is another limitation; immune cells may have moved between *in-vivo* and *ex-vivo* imaging, leading to misalignment following vessel-based registration. It is also the case that since mice were only culled after *in-vivo* imaging on day 10, the molecular content of the features observed in the *in-vivo* imaging on day 3 and day 7 could not be elucidated. A further limitation is the two-dimensional registration procedure implemented here. Fiducial points selected from within the deep capillary plexus (DCP), in both the *in-vivo* and *ex-vivo* images, form the basis of the registration. Since retinal tissue is prone to distortion when flat-mounted for confocal imaging, 2D deformation fields calculated at a particular retinal layer (here the DCP) are unlikely to be effective for alignment at different depths within the tissue. The effects of this can be seen in the slight misalignment of features in the upper region of the DOPU-IB4 overlay in Fig. 5. As opposed to the cell circled in blue, which sits within the DCP layer, these hyperreflective features are positioned slightly below the DCP. The result is that the deformation fields calculated at the DCP do not satisfactorily align the features in the IB4 channel and the DOPU signal.

Future work will involve further validation of our findings with the potential for eventual translation to clinical studies. A greater sample size is required to confirm our findings. Three-dimensional registration may alleviate misalignment caused by distortion of the retinal tissue when prepared for *ex-vivo* confocal imaging. Cardiac perfusion should be performed immediately after *in-vivo* imaging to ensure the cellular alignment is as close as possible between *in-vivo* and *ex-vivo* imaging.

## Supplemental information

Supplement 1 Supplemental figure https://doi.org/10.6084/m9.figshare.32234730

## Data Availability

Data underlying the results presented in this paper are not publicly available at this time.

## Supplemental document

See Supplement 1 for supporting content.

## References

[1] Huang D. Swanson E. A. Lin C. P. et al., “Optical Coherence Tomography,” Science 254(5035), 1178–1181 (1991). 10.1126/science.1957169 1957169 PMC4638169

[2] Fujimoto J. Swanson E., “The Development, Commercialization, and Impact of Optical Coherence Tomography,” Invest. Ophthalmol. Vis. Sci. 57(9), OCT1 (2016). 10.1167/iovs.16-19963 27409459 PMC4968928

[3] Fragiotta S. Abdolrahimzadeh S. Dolz-Marco R. et al., “Significance of Hyperreflective Foci as an Optical Coherence Tomography Biomarker in Retinal Diseases: Characterization and Clinical Implications,” J. Ophthalmol. 2021, 1–10 (2021). 10.1155/2021/6096017 PMC8709761 34956669

[4] Nassisi M. Lei J. Abdelfattah N. S. et al., “OCT Risk Factors for Development of Late Age-Related Macular Degeneration in the Fellow Eyes of Patients Enrolled in the HARBOR Study,” Ophthalmology 126(12), 1667–1674 (2019). 10.1016/j.ophtha.2019.05.016 31281056

[5] Rübsam A. Wernecke L. Rau S. et al., “Behavior of SD-OCT Detectable Hyperreflective Foci in Diabetic Macular Edema Patients after Therapy with Anti-VEGF Agents and Dexamethasone Implants,” Journal of Diabetic Research 2021, 1–13 (2021). 10.1155/2021/8820216 PMC8060103 33937416

[6] Saito M. Barbazetto I. A. Spaide R. F., “Intravitreal Cellular Infiltrate Imaged As Punctate Spots by Spectral-Domain Optical Coherence Tomography In Eyes With Posterior Segment Inflammatory Disease,” Retina 33(3), 559–565 (2013). 10.1097/IAE.0b013e31826710ea 23042101

[7] Cao D. Leong B. Messinger J. D. et al., “Hyperreflective Foci, Optical Coherence Tomography Progression Indicators in Age-Related Macular Degeneration, Include Transdifferentiated Retinal Pigment Epithelium,” Invest. Ophthalmol. Visual Sci. 62(10), 34 (2021). 10.1167/iovs.62.10.34 PMC8399556 34448806

[8] Nassisi M. Fan W. Shi Y. et al., “Quantity of Intraretinal Hyperreflective Foci in Patients With Intermediate Age-Related Macular Degeneration Correlates With 1-Year Progression,” Invest. Ophthalmol. Vis. Sci. 59(8), 3431 (2018). 10.1167/iovs.18-24143 30025092

[9] Boer J. F. d. Hitzenberger C. K. Yasuno Y., “Polarization sensitive optical coherence tomography,” Biomed. Opt. Express 8(3), 1838–1873 (2017). 10.1364/BOE.8.001838 28663869 PMC5480584

[10] Schmitt J. M. Xiang S. H., “Cross-polarized backscatter in optical coherence tomography of biological tissue,” Opt. Lett. 23(13), 1060–1062 (1998). 10.1364/OL.23.001060 18087429

[11] Haskell R. C. Carlson F. D. Blank P. S., “Form birefringence of muscle,” Biophys. J. 56(2), 401–413 (1989). 10.1016/S0006-3495(89)82686-4 2775834 PMC1280489

[12] Baumann B. Baumann S. O. Konegger T. et al., “Polarization sensitive optical coherence tomography of melanin provides intrinsic contrast based on depolarization,” Biomed. Opt. Express 3(7), 1670–1683 (2012). 10.1364/BOE.3.001670 22808437 PMC3395490

[13] Pircher M. Zawadzki R. J., “Review of adaptive optics OCT (AO-OCT): principles and applications for retinal imaging,” Biomed. Opt. Express 8(5), 2536–2562 (2017). 10.1364/BOE.8.002536 28663890 PMC5480497

[14] Cense B. Gao W. Brown J. M. et al., “Retinal imaging with polarization-sensitive optical coherence tomography and adaptive optics,” Opt. Express 17(24), 21634–21651 (2009). 10.1364/OE.17.021634 19997405 PMC3113602

[15] Kurokawa K. Nemeth M., “Multifunctional adaptive optics optical coherence tomography allows cellular scale reflectometry, polarimetry, and angiography in the living human eye,” Biomed. Opt. Express 15(2), 1331–1354 (2024). 10.1364/BOE.505395 38404344 PMC10890865

[16] Hsu D. Kwon J. H. Ng R. et al., “Quantitative multi-contrast in vivo mouse imaging with polarization diversity optical coherence tomography and angiography,” Biomed. Opt. Express 11(12), 6945–6961 (2020). 10.1364/BOE.403209 33408972 PMC7747897

[17] Song J. Hu Y. Chen A. et al., “In vivo multi-contrast depth-resolved choroidal imaging of a mouse using polarization-diversity optical coherence tomography,” Opt. Lett. 49(15), 4314–4317 (2024). 10.1364/OL.529146 39090922

[18] Fan W. Miller D. A. Chang S. et al., “Longitudinal imaging of vitreal hyperreflective foci in mice with acute optic nerve damage using visible-light optical coherence tomography,” Opt. Lett. 49(8), 1880–1883 (2024). 10.1364/OL.512029 38621029 PMC11217911

[19] Nor M. N. M. Guo C. X. Green C. R. et al., “Hyper-reflective dots in optical coherence tomography imaging and inflammation markers in diabetic retinopathy,” J. Anat. 243(4), 697–705 (2023). 10.1111/joa.13889 37222261 PMC10485584

[20] Miller E. B. Zhang P. Ching K. et al., “In vivo imaging reveals transient microglia recruitment and functional recovery of photoreceptor signaling after injury,” Proc. Natl. Acad. Sci. 116(33), 16603–16612 (2019). 10.1073/pnas.1903336116 31350349 PMC6697899

[21] Wahl D. Ng R. Ju M. et al., “Sensorless adaptive optics multimodal enface small animal retinal imaging,” Biomed. Opt. Express 10(1), 252–266 (2019). 10.1364/BOE.10.000252 30775098 PMC6363194

[22] Wahl D. Jian Y. Bonora S. et al., “Wavefront sensorless adaptive optics fluorescence biomicroscope for in vivo retinal imaging in mice,” Biomed. Opt. Express 7(1), 1–12 (2016). 10.1364/BOE.7.000001 26819812 PMC4722895

[23] Miao Y. Song J. Hsu D. et al., “Numerical calibration method for a multiple spectrometer-based OCT system,” Biomed. Opt. Express 13(3), 1685–1701 (2022). 10.1364/BOE.450942 35414988 PMC8973183

[24] Götzinger E. Baumann B. Pircher M. et al., “Polarization maintaining fiber based ultra-high resolution spectral domain polarization sensitive optical coherence tomography,” Opt. Express 17(25), 22704–22717 (2009). 10.1364/OE.17.022704 20052196 PMC2963062

[25] Götzinger E. Pircher M. Geitzenauer W. et al., “Retinal pigment epithelium segmentation by polarization sensitive optical coherence tomography,” Opt. Express 16(21), 16410–16422 (2008). 10.1364/OE.16.016410 18852747 PMC2976032

[26] Makita S. Hong Y.-J. Miura M. et al., “Degree of polarization uniformity with high noise immunity using polarization-sensitive optical coherence tomography,” Opt. Lett. 39(24), 6783–6786 (2014). 10.1364/OL.39.006783 25502996

[27] Mariampillai A. Standish B. A. Moriyama E. H. et al., “Speckle variance detection of microvasculature using swept-source optical coherence tomography,” Opt. Lett. 33(13), 1530–1532 (2008). 10.1364/OL.33.001530 18594688

[28] Mariampillai A. Leung M. K. K. Jarvi M. et al., “Optimized speckle variance OCT imaging of microvasculature,” Opt. Lett. 35(8), 1257–1259 (2010). 10.1364/OL.35.001257 20410985

[29] Esper M. E. Kodippili K. Rudnicki M. A., “Immunofluorescence Labeling of Skeletal Muscle in Development, Regeneration, and Disease,” Histochemistry of Single Molecules 2566, 113–132 (2023). 10.1007/978-1-0716-2675-7_9 PMC10204082 36152246

[30] Fialová S. Augustin M. Glösmann M. et al., “Polarization properties of single layers in the posterior eyes of mice and rats investigated using high resolution polarization sensitive optical coherence tomography,” Biomed. Opt. Express 7(4), 1479–1495 (2016). 10.1364/BOE.7.001479 27446670 PMC4929656

[31] Giani A. Thanos A. Roh M. I. et al., “In Vivo Evaluation of Laser-Induced Choroidal Neovascularization Using Spectral-Domain Optical Coherence Tomography,” Invest. Ophthalmol. Vis. Sci. 52(6), 3880 (2011). 10.1167/iovs.10-6266 21296820

[32] Hou K. K. Au A. Kashani A. H. et al., “Pseudoflow with OCT Angiography in Eyes with Hard Exudates and Macular Drusen,” Trans. Vis. Sci. Tech. 8(3), 50 (2019). 10.1167/tvst.8.3.50 PMC6601711 31293805

[33] Wilk M. A. Huckenpahler A. L. Collery R. F. et al., “The Effect of Retinal Melanin on Optical Coherence Tomography Images,” Trans. Vis. Sci. Tech. 6(2), 8 (2017). 10.1167/tvst.6.2.8 PMC5381330 28392975

