# Supplementary material for: Histology correlated adaptive optics polarisation sensitive optical coherence tomography
Source: Biomed Opt Express. 2026 May 19;17(6):3059–71. doi: 10.1364/BOE.590238 (PMC13271213; doi:10.1364/BOE.590238)
Supplement: Supplementary file 1 [file boe-17-6-3059-s001.pdf]

Supplemental Document

Biomedical Optics EXPRESS

# Histology correlated adaptive optics polarisation sensitive optical coherence tomography: supplement

THOMAS J. SMART,[1,*] BRUNO CHARBIT,[1] ZIQI ZHOU,[2] YUAN TIAN,[1] ARMAN ATHWAL,[2] JUN SONG,[3] MYEONG JIN JU,[3,4] COLIN J. CHU,[1,5] AND MARINKO V. SARUNIC[1,2,6]

[1] *Institute of Ophthalmology, University College London, London EC1V 9EL, United Kingdom*
[2] *Department of Medical Physics and Biomedical Engineering, University College London, London WC1E 6BT, UK*
[3] *School of Biomedical Engineering, University of British Columbia, Vancouver, BC, Canada*
[4] *Department of Ophthalmology and Visual Sciences, University of British Columbia, Vancouver, BC, Canada*
[5] *Moorfields Eye Hospital, London, London EC1V 2PD, United Kingdom*
[6] *School of Engineering Science, Simon Fraser University, Burnaby BC V5A 1S6, Canada*
[*] *thomas.smart@ucl.ac.uk*

This supplement published with Optica Publishing Group on 19 May 2026 by The Authors under the terms of the Creative Commons Attribution 4.0 License in the format provided by the authors and unedited. Further distribution of this work must maintain attribution to the author(s) and the published article's title, journal citation, and DOI.

Supplement DOI: https://doi.org/10.6084/m9.figshare.32234730

Parent Article DOI: https://doi.org/10.1364/BOE.590238

Figure S1 shows an example of a hyperreflective foci (HRF) in the OCT reflectance modality with a low contrast counterpart in the DOPU modality. The HRF shown here is positioned peripherally and superficially relative to lesion in figures 4 and 5 of the manuscript. Subfigure S1A shows the HRF (circled in magenta) in the OCT reflectance channel in orthogonal (B-scan) and *en-face* planes. The red box in the B-scan indicates the depth and *x,y* region of the *en-face* projection*.* S1B shows the same region in the DOPU modality. Whilst the HRF is just visible in the DOPU, the contrast is significantly lower compared to the HRF highlighted in Figure 5 of the manuscript.

Subfigure S1C shows the IB4 channel (in red) registered to and overlayed on the OCT reflectance. The OCT is now shown in green to permit visualisation of co-localisation. The yellow colouration within the magenta circle indicates co-localisation of the IB4-labelled cell and the HRF in the OCT reflectance channel. A magnified view of the IB4-labelled cell is shown along with the confocal transmission channel. Unlike the cell highlighted in figure 5, there is no accompanying dark region in the transmission channel.

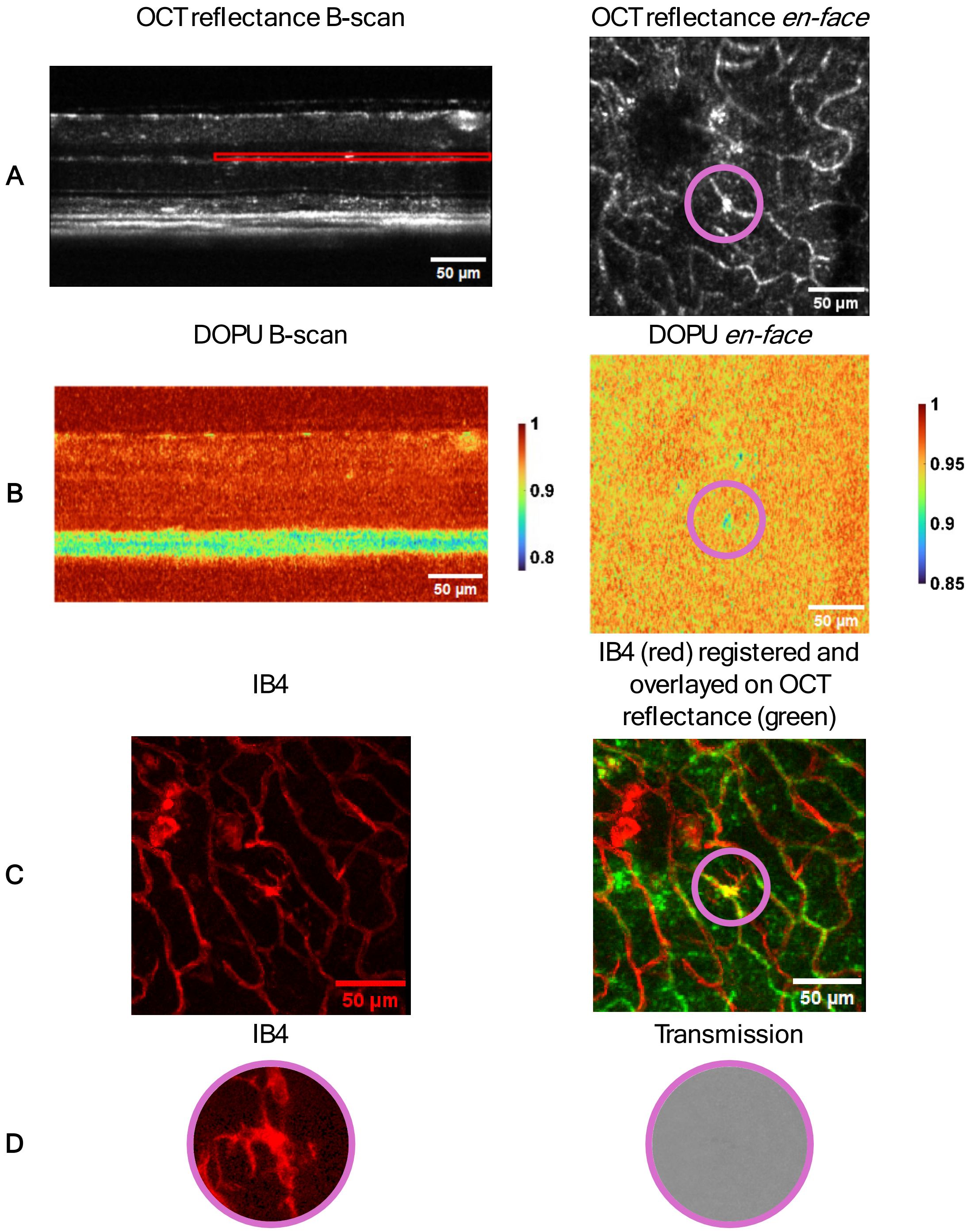


Figure S1. Example of an HRF with strong OCT reflectance and weak DOPU signal. **A:** B-scan and en-face projection in OCT reflectance. The red box on the B-scan indicates the depth and extent of the *x,y* region in the *en-face* projection. Magenta circle in the *en-face* image highlights HRF **B:** B-scan and en-face projection in DOPU contrast. **C:** IB4 registered and overlayed on OCT en-face. The OCT reflectance is shown in green to allow visualisation of co-localisation, see yellow colouration within magenta circle. **D:** magnified images from IB4 and confocal transmission channels from within magenta circle.
